# Regulation of De Novo Lipid Synthesis by the Small GTPase Rac1 in the Adipogenic Differentiation of Progenitor Cells from Mouse White Adipose Tissue

**DOI:** 10.3390/ijms24054608

**Published:** 2023-02-27

**Authors:** Kiko Hasegawa, Nobuyuki Takenaka, Maaya Yamamoto, Yoshiki Sakoda, Atsu Aiba, Takaya Satoh

**Affiliations:** 1 Laboratory of Cell Biology, Department of Biological Chemistry, Graduate School of Science, Osaka Metropolitan University, Sakai 599-8531, Osaka, Japan; 2 Laboratory of Animal Resources, Center for Disease Biology and Integrative Medicine, Graduate School of Medicine, The University of Tokyo, Bunkyo-ku, Tokyo 113-0033, Japan

**Keywords:** adipogenic differentiation, adipose progenitor cell, C/EBPα, GTPase, insulin, lipogenic enzymes, PPARγ, obesity, Rac1, RalA, white adipocyte

## Abstract

White adipocytes act as lipid storage, and play an important role in energy homeostasis. The small GTPase Rac1 has been implicated in the regulation of insulin-stimulated glucose uptake in white adipocytes. Adipocyte-specific *rac1*-knockout (adipo-*rac1*-KO) mice exhibit atrophy of subcutaneous and epididymal white adipose tissue (WAT); white adipocytes in these mice are significantly smaller than controls. Here, we aimed to investigate the mechanisms underlying the aberrations in the development of Rac1-deficient white adipocytes by employing in vitro differentiation systems. Cell fractions containing adipose progenitor cells were obtained from WAT and subjected to treatments that induced differentiation into adipocytes. In concordance with observations in vivo, the generation of lipid droplets was significantly attenuated in Rac1-deficient adipocytes. Notably, the induction of various enzymes responsible for de novo synthesis of fatty acids and triacylglycerol in the late stage of adipogenic differentiation was almost completely suppressed in Rac1-deficient adipocytes. Furthermore, the expression and activation of transcription factors, such as the CCAAT/enhancer-binding protein (C/EBP) β, which is required for the induction of lipogenic enzymes, were largely inhibited in Rac1-deficient cells in both early and late stages of differentiation. Altogether, Rac1 is responsible for adipogenic differentiation, including lipogenesis, through the regulation of differentiation-related transcription.

## 1. Introduction

WAT is a type of mammalian adipose tissue serving as storage of lipids, and plays a pivotal role in energy and glucose homeostasis [1]. Triacylglycerol is the main constituent of lipid droplets in white adipocytes, and is synthesized from glucose and fatty acids through multiple metabolic reactions. On the other hand, the transport of glucose and fatty acids into adipocytes from the blood is mediated by various specific transporters. Insulin is known to enhance the synthesis and accumulation of triacylglycerol, as well as glucose and fatty acid uptake in adipocytes.

The glucose transporter GLUT4 is responsible for insulin-stimulated glucose uptake in adipocytes [2]. The increase in the level of plasma membrane-localized GLUT4 in response to insulin results in the enhanced uptake of glucose into the cell [2,3]. Signal-transducing pathways downstream of the insulin receptor leading to GLUT4 translocation to the plasma membrane in adipocytes have been investigated extensively for decades, and two major signaling pathways are well characterized [3,4]. One signaling cascade comprises phosphoinositide 3-kinase (PI3K) and the serine/threonine protein kinases PDK1 and Akt2. This signaling cascade plays a crucial role in both adipocytes and skeletal muscle [3,4,5]. Downstream of Akt2, the Akt substrate of 160 kDa, also termed TBC1D4, has been implicated in the regulation of GLUT4 vesicle trafficking in response to insulin, acting as a GTPase-activating protein for the Rab family small GTPase Rab10 in adipocytes [6,7,8]. The other cascade is thought to be specific to adipocytes and independent of PI3K [9].

In addition, the Rho family small GTPase Rac1 has been implicated in insulin-stimulated glucose uptake in primary cultured mouse adipocytes and differentiated mouse 3T3-L1 adipocytes [10,11,12,13]. The guanine nucleotide exchange factor FLJ00068, which is responsible for Akt2-dependent Rac1 activation in skeletal muscle insulin signaling [14,15], was shown to act as a regulator for Rac1 downstream of Akt2 in 3T3-L1 adipocytes [12]. Another small GTPase, RalA, which belongs to the Ras family, also plays an important role in insulin-stimulated GLUT4 translocation [10,11,16,17,18,19]. Considering that RalA is regulated downstream of Rac1 in skeletal muscle insulin signaling [20,21], it is possible that RalA acts downstream of Rac1 in adipocytes as well. Indeed, we demonstrated that RalA regulates GLUT4 translocation downstream of Rac1 in 3T3-L1 adipocytes [11].

To further determine the physiological role of Rac1, we generated mice lacking Rac1 specifically in adipose tissue, adipo-*rac1*-KO mice [13]. Subcutaneous and epididymal WAT in adipo-*rac1*-KO mice were significantly smaller than those in wild-type mice. Correspondingly, white adipocytes that lacked Rac1 were smaller than controls [13]. Glucose uptake and GLUT4 translocation in response to insulin were reduced in *rac1*-KO white adipocytes [13]. In addition, the expression of various enzymes for fatty acid and triacylglycerol synthesis [22,23], including ATP citrate lyase (ACLY), acetyl-CoA carboxylase (ACC), fatty acid synthase (FASN), stearoyl-CoA desaturase 1 (SCD1), and glycerol-3-phosphate acyltransferase 1 (GPAT1), were downregulated in white adipocytes of adipo-*rac1*-KO mice [13]. Thus, we propose that Rac1 is involved in de novo synthesis of lipids as well as glucose uptake in white adipocytes, regulating hypertrophy of WAT.

The expression of the enzymes for the synthesis of fatty acids and triacylglycerol is known to be regulated by a variety of transcription factors, such as the nuclear receptor peroxisome proliferator-activated receptor γ (PPARγ) [24] and CCAAT/enhancer-binding protein (C/EBP) family transcription factors [25], in response to insulin. Thus, it is important to examine expression levels of these transcription factors to clarify the role of Rac1 in the regulation of de novo lipid synthesis in white adipocytes.

In this study, we aimed to further reveal the mechanisms underlying atrophy of WAT in adipo-*rac1*-KO mice, employing in vitro differentiation systems of mouse progenitor cells isolated from WAT and the 3T3-L1 cell line. We show that Rac1 plays a pivotal role in the induction of differentiation into adipocytes, regulating not only glucose uptake but also the expression of diverse enzymes for de novo lipid synthesis.

## 2. Results

### 2.1. Establishment and Characterization of an In Vitro Differentiation Assay Using Adipose Progenitor Cells Obtained from Mouse WAT

**Figure 1:**
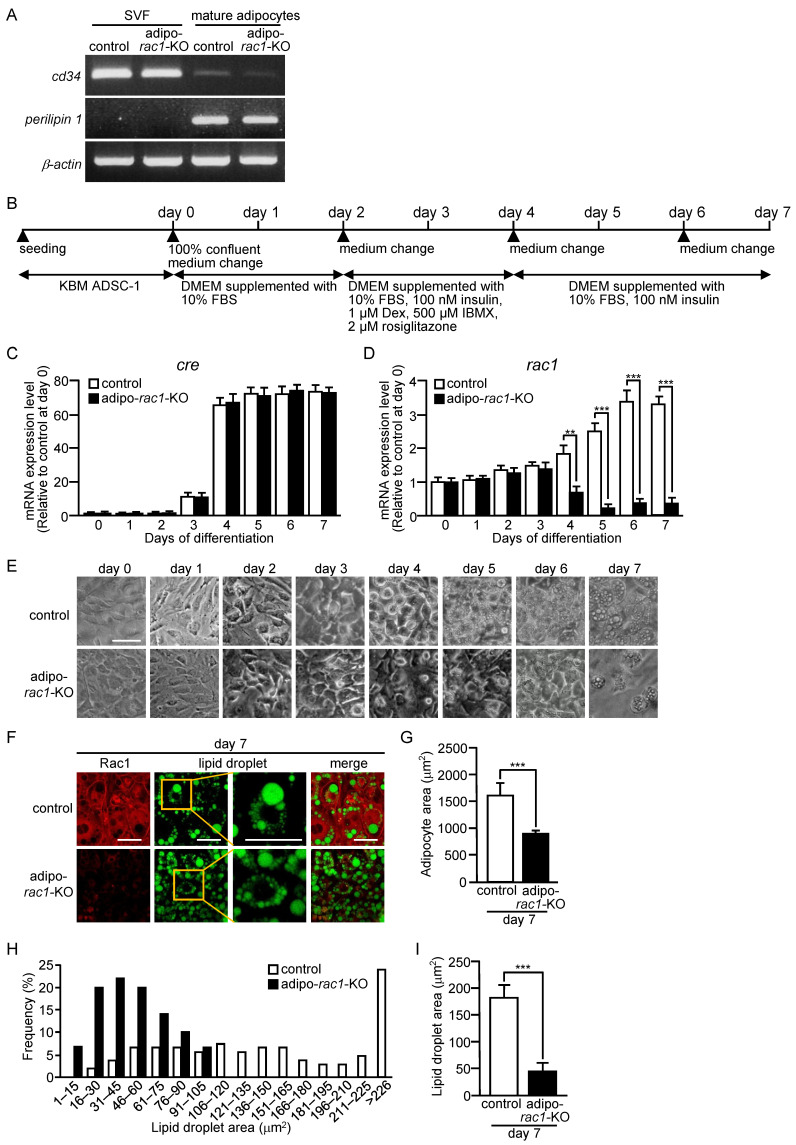
Differentiation of adipose progenitor cells obtained from mouse WAT into adipocytes in vitro. (**A**) The expression of *cd34* and *perilipin 1* genes in the SVF and mature adipocytes. Relative mRNA levels were determined by RT-PCR analysis. The *β-actin* gene served as a loading control. (**B**) Schematic representation of the differentiation protocol. The day when cells reached confluence was referred to as day 0. (**C**) The expression of the *cre* gene during adipogenic differentiation in vitro. Relative mRNA levels in cells derived from control and adipo-*rac1*-KO mice were determined by quantitative RT-PCR analysis. Data are shown as means ± S.E. (*n* = 3). (**D**) The expression of the *rac1* gene during adipogenic differentiation in vitro. Relative mRNA levels in cells derived from control and adipo-*rac1*-KO mice were determined by quantitative RT-PCR analysis. Data are shown as means ± S.E. (*n* = 3). ** *p* < 0.01, *** *p* < 0.001 (Student’s *t* test). (**E**) Phase-contrast images of cells derived from control and adipo-*rac1*-KO mice during adipogenic differentiation in vitro. Scale bar, 50 μm. (**F**) Fluorescent staining of Rac1 and lipid droplets with an anti-Rac1 antibody and LipiDye, respectively, in differentiated adipocytes at day 7. For the staining of lipid droplets, the high-magnification image (right) of the boxed area in the low magnification image (left) is also shown. Scale bar, 25 μm. (**G**) Areas of differentiated adipocytes at day 7. Data are shown as means ± S.E. (*n* = 100). *** *p* < 0.001 (Student’s *t* test). (**H**) Histograms depicting the distribution of areas of lipid droplets in differentiated adipocytes at day 7. Data were obtained from 100 lipid droplets in 5 images. (**I**) Areas of lipid droplets in differentiated adipocytes at day 7. Data are shown as means ± S.E. (*n* = 100). *** *p* < 0.001 (Student’s *t* test).

We established an in vitro differentiation assay as a first step to clarify the mechanisms for atrophy of WAT observed in adipo-*rac1*-KO mice [13]. Collagenase-treated mouse subcutaneous WAT was centrifuged and mature adipocytes as floating cells were removed. We then confirmed that the precipitated stromal vascular fraction (SVF) contained CD34-positive adipose progenitor cells, but not perilipin 1-positive mature adipocytes, by reverse-transcriptase polymerase chain reaction (RT-PCR) analysis [26,27] (Figure 1A).

The SVF containing adipose progenitor cells was cultured until cells reached confluence in a culture medium optimized for growth of adipose progenitor cells (KBM ADSC-1) (Figure 1B). The day when cells reached confluence was referred to as day 0. Confluent cells were further cultured for two days in Dulbecco’s modified Eagle’s medium (DMEM)-based growth medium and then subjected to treatment with reagents, such as insulin, which are required for differentiation into adipocytes, as described in Figure 1B.

Expression levels of genes for the Cre recombinase and Rac1 during adipogenic differentiation in vitro from progenitor cells were monitored by quantitative RT-PCR analysis (Figure 1C,D). The Cre recombinase transgene is expressed under the control of the adiponectin (Adipoq) promoter, which is specifically activated in adipocytes [28]. Therefore, the expression of the Cre recombinase transgene was expected to increase after the initiation of adipogenic differentiation. In fact, the expression of the Cre recombinase transgene in cells derived from control (adipo-Cre) and adipo-*rac1*-KO mice was stimulated after 3-day induction of adipogenic differentiation, and reached a plateau at day 4 (Figure 1C). Consistently with these observations, the expression level of the *rac1* gene was significantly reduced after 4-day induction of adipogenic differentiation in cells derived from adipo-*rac1*-KO mice (Figure 1D). The expression of the *rac1* gene was enhanced after day 5 in control cells, suggesting a significant role of Rac1 in differentiated adipocytes.

After 4-day induction of adipogenic differentiation, small lipid droplets emerged in cells derived from control mice (Figure 1E). In contrast, virtually no cells derived from adipo-*rac1*-KO mice contained lipid droplets in this stage (Figure 1E). At day 7, a large quantity of lipid droplets was detected in cells from control mice, whereas only a limited number of cells from adipo-*rac1*-KO mice contained lipid droplets (Figure 1E). Differentiated adipocytes harboring lipid droplets were collected by centrifugation from the cell cultures at day 7, and seeded onto chamber slides. The Rac1 protein and lipid droplets were then stained with an anti-Rac1 antibody and a fluorescent dye for lipid droplets, respectively (Figure 1F). The average size of cells from adipo-*rac1*-KO mice was significantly less than that of control cells (Figure 1G). The size of lipid droplets in cells from adipo-*rac1*-KO mice was also measured, and found to be largely reduced compared with that in control cells (Figure 1H,I). Taken together, these results demonstrate that knockdown of the *rac1* gene during the process of adipogenic differentiation severely affects the accumulation of lipid droplets in adipocytes. These results also show that the effect of Rac1 deficiency on adipogenic differentiation in vivo is well reproduced by the aforementioned in vitro differentiation assay using adipose progenitor cells.

### 2.2. The Expression of Enzymes for De Novo Synthesis of Fatty Acids and Triacylglycerol during Differentiation of Adipose Progenitor Cells into Adipocytes In Vitro

**Figure 2:**
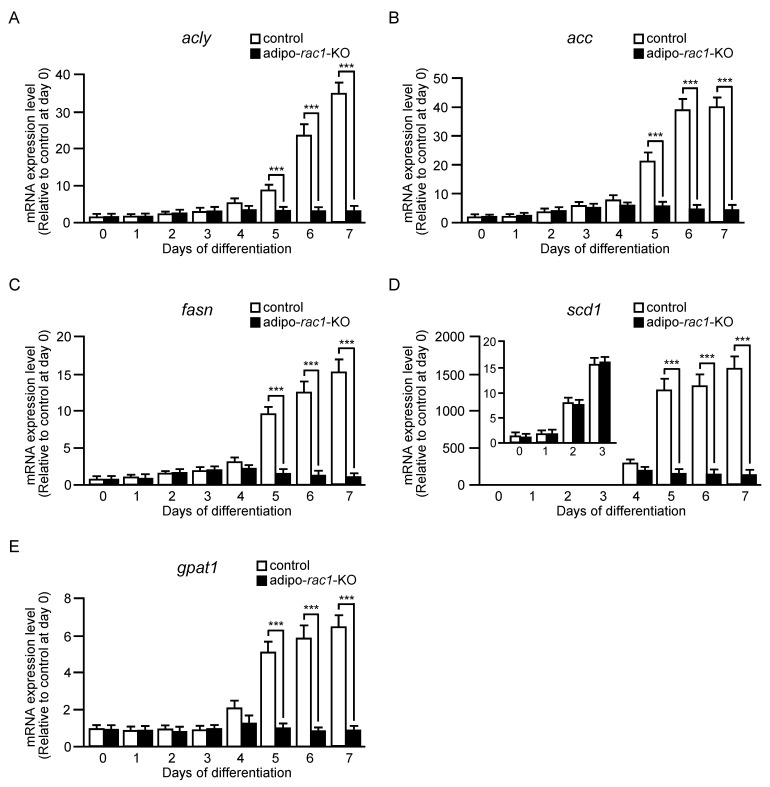
The expression of enzymes for de novo synthesis of fatty acids and triacylglycerol during differentiation of adipose progenitor cells into adipocytes in vitro. The expression of *acly* (**A**), *acc* (**B**), *fasn* (**C**), *scd1* (**D**), and *gpat1* (**E**) genes during adipogenic differentiation in vitro. Relative mRNA levels in cells derived from control and adipo-*rac1*-KO mice were determined by quantitative RT-PCR analysis. Data are shown as means ± S.E. (*n* = 3). *** *p* < 0.001 (Student’s *t* test).

We previously demonstrated that mRNA levels of various enzymes for de novo synthesis of fatty acids and triacylglycerol, such as ACLY, ACC, FASN, SCD1, and GPAT1, were significantly lowered in subcutaneous WAT in adipo-*rac1*-KO mice [13]. These results suggest that Rac1 is responsible for the regulation of de novo lipid synthesis [13]. To further explore this possibility, we next assessed expression levels of the above enzymes during adipogenic differentiation in vitro. In the control cell culture, the expression of the genes that encode the above enzymes was highly stimulated during adipogenic differentiation (Figure 2). The increase in the expression level of the gene encoding ACLY was observed at day 5, followed by further increase until day 7 (Figure 2A). Likewise, expression levels of genes encoding ACC, FASN and GPAT1 started to increase at day 5, and continued to increase up to the maximal level around day 6 (Figure 2B,C,E). The expression level of the *scd1* gene was increased after day 2, and reached a maximum at day 5 (Figure 2D). In marked contrast, virtually no increase in expression levels of all of the above genes was detected in cells derived from adipo-*rac1*-KO mice (Figure 2). These results provide evidence that Rac1 is intimately involved in the induction of genes that encode enzymes for de novo synthesis of fatty acids and triacylglycerol during adipogenic differentiation. The expression of the *scd1* gene was increased until day 3 not only in control cells but also in cells from adipo-*rac1*-KO mice, because the *rac1* gene was not disrupted at this time point (Figure 2D).

### 2.3. The Expression and Phosphorylation of Transcription Factors during Differentiation of Adipose Progenitor Cells into Adipocytes In Vitro

**Figure 3:**
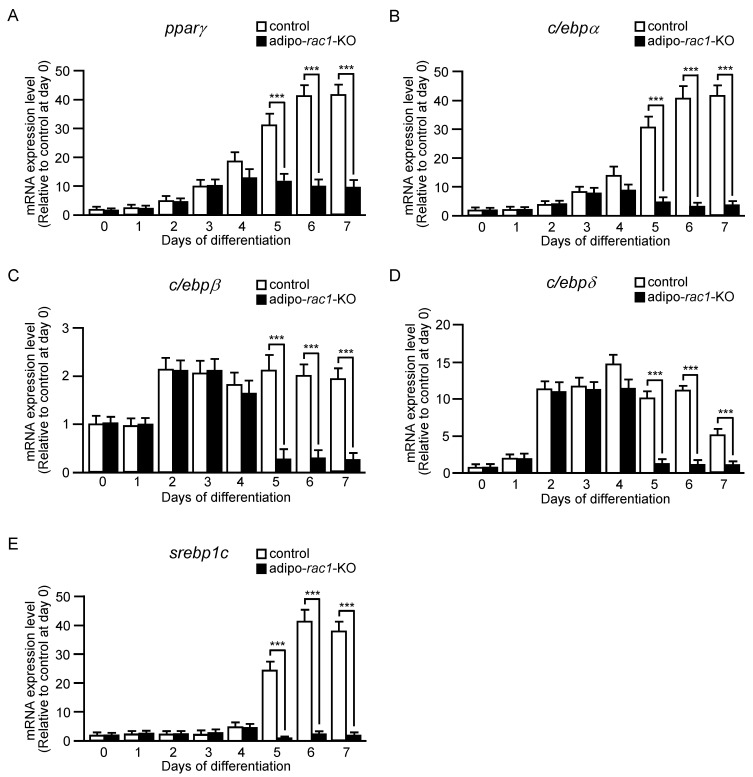
The expression of transcription factors during differentiation of adipose progenitor cells into adipocytes in vitro. The expression of *pparγ* (**A**), *c/ebpα* (**B**), *c/ebpβ* (**C**), *c/ebpδ* (**D**), *srebp1c* (**E**) genes during adipogenic differentiation in vitro. Relative mRNA levels in cells derived from control and adipo-*rac1*-KO mice were determined by quantitative RT-PCR analysis. Data are shown as means ± S.E. (*n* = 3). *** *p* < 0.001 (Student’s *t* test).

To further explore the role of Rac1 in the induction of adipogenic differentiation, we next examined the expression of various transcription factors that have been implicated in the upregulation of differentiation-related genes. PPARγ activates a variety of target genes, regulating adipocyte differentiation and function [24]. The expression level of the gene encoding PPARγ started to increase at day 2, and reached a maximum at day 6 in control cells (Figure 3A). In cells derived from adipo-*rac1*-KO mice, this gene was induced until day 3, but virtually no additional increase was observed after day 4, at which point the expression of the *rac1* gene was suppressed (Figure 3A). Therefore, it is likely that Rac1 is involved in the induction of the gene encoding PPARγ during adipogenic differentiation.

Another family of transcription factors, the C/EBP family, is composed of six members, in which the basic leucine zipper (bZIP) domain is conserved at the C terminus [25]. Among them, C/EBPα has been implicated in the control of differentiation into adipocytes through the induction of diverse target genes. The mRNA level of C/EBPα in control cells was increased as adipogenic differentiation proceeded, whereas no induction was observed after day 4 in cells isolated from adipo-*rac1*-KO mice, similarly to the case of PPARγ (Figure 3B). The expression of genes for C/EBPβ and C/EBPδ isoforms is known to be promoted in the early stage of adipogenic differentiation, and these two isoforms are involved in the induction of PPARγ and C/EBPα [25]. C/EBPβ and C/EBPδ mRNAs were increased to near-maximal levels within two days in cells from both control and adipo-*rac1*-KO mice (Figure 3C,D). Expression levels of C/EBPβ and C/EBPδ in cells from adipo-*rac1*-KO mice were largely decreased after 5-day induction of differentiation, in contrast to control cells, in which the expression levels were sustained (Figure 3C,D).

Sterol regulatory element-binding protein 1c (SREBP-1c) is a transcription factor implicated in the regulation of fatty acid and triacylglycerol synthesis in the liver and adipose tissue [23,29]. We next examined mRNA levels of SREBP-1c in cells from control and adipo-*rac1*-KO mice, because SREBP-1c activates genes encoding enzymes for de novo lipid synthesis described above in response to insulin [23,29] (Figure 3E). The mRNA level of SREBP-1c was rapidly increased at day 5 and sustained until day 7 in control cells. In contrast, virtually no increase in the mRNA level was observed in cells derived from adipo-*rac1*-KO mice.

**Figure 4:**
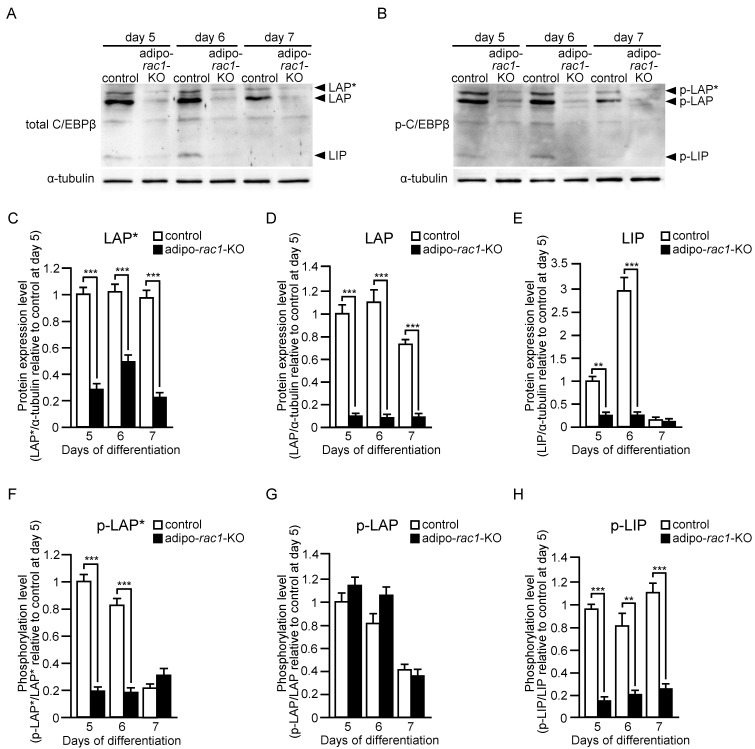
Protein and phosphorylation levels of three C/EBPβ variants in the late stage of differentiation of adipose progenitor cells into adipocytes in vitro. Protein (**A**) and phosphorylation (**B**) levels of three C/EBPβ variants (LAP*, LAP, and LIP) in cells derived from control and adipo-*rac1*-KO mice were determined by immunoblot analysis using antibodies against C/EBPβ (**A**) and phosphorylated C/EBPβ (**B**), respectively. α-tubulin served as a loading control. Intensities of bands for LAP* (**C**), LAP (**D**), LIP (**E**), phosphorylated LAP* (p-LAP*) (**F**), phosphorylated LAP (p-LAP) (**G**), and phosphorylated LIP (p-LIP) (**H**) in immunoblots were quantified. Data are shown as means ± S.E. (*n* = 3). ** *p* < 0.01, *** *p* < 0.001 (Student’s *t* test).

Three different-sized polypeptides, named LAP*, LAP, and LIP, are produced from the C/EBPβ mRNA molecule by alternative use of translation initiation codons [25]. Both transcriptional activation and bZIP domains are present in LAP* and LAP, whereas LIP contains the bZIP domain, but not the transcriptional activation domain. Therefore, LIP is thought to act as a dominant-negative form by dimerizing with LAP* or LAP [25]. Furthermore, sequential phosphorylation of C/EBPβ by mitogen-activated protein kinase, cyclin-dependent kinase 2/cyclin A, and glycogen synthase kinase 3β increases the DNA-binding activity [30]. We then examined protein and phosphorylation levels of LAP*, LAP, and LIP by immunoblot analysis in the late stage of differentiation (Figure 4). Protein levels of LAP* and LAP were sustained from day 5 to day 7 in control cells, whereas the levels in cells derived from adipo-*rac1*-KO mice were markedly reduced similarly to the mRNA levels (Figure 4A,C,D). The protein level of LIP in cells from control mice was increased at day 6, but rapidly decreased at day 7 (Figure 4A,E). Although the mechanisms for this change remain unclear, the rapid decrease at day 7 may contribute to further induction of target genes. In cells from adipo-*rac1*-KO mice, the protein level of LIP was also largely suppressed, but its effect on the induction of target genes may be limited due to the low levels of LAP* and LAP (Figure 4A,E). Phosphorylation levels of the above three variants of C/EBPβ were also evaluated by using a phospho-specific antibody. Phosphorylation levels of LAP* and LAP in control cells rapidly decreased at day 7, suggesting lowered transcription activities of these proteins at this point (Figure 4B,F,G). The phosphorylation level of LAP* was largely suppressed in adipo-*rac1*-KO mice-derived cells from day 5 to day 6, suggesting a role of Rac1 in the regulation of phosphorylation (Figure 4B,F). In contrast, the phosphorylation level of LAP in cells from adipo-*rac1*-KO mice was similar to that in control cells (Figure 4B,G). The phosphorylation level of LIP was also significantly reduced in cells from adipo-*rac1*-KO mice (Figure 4B,H).

### 2.4. Protein and Phosphorylation Levels of C/EBPβ in the Early Stage of Differentiation of Adipose Progenitor Cells into Adipocytes In Vitro

The induced expression of the *rac1* gene after day 5 in control cells suggested an important role of Rac1 in the late stage of differentiation (Figure 1D), and indeed Rac1 was involved in the regulation of lipogenesis in this stage, as described above. It is also important to clarify the function of Rac1 in the early stage of differentiation, because the *rac1* gene was significantly expressed (approximately 30% of the maximal level) even before day 3 (Figure 1D), and the activity of Rac1 is generally enhanced through GDP/GTP exchange (GTP-binding) of preexisting Rac1 molecules rather than induced expression [10]. Therefore, we next tested the effect of functional inactivation of Rac1 on the expression and activation of C/EBPβ in the early stage. We cannot examine whether Rac1 is required for adipogenic differentiation before day 3 by Cre-mediated knockdown of the *rac1* gene in the in vitro differentiation system using adipose progenitor cells from mouse WAT, because knockdown of Rac1 was initiated at day 4 (Figure 1D). Thus, we employed two types of specific chemical inhibitors of Rac1, RI-II and NSC23766, to address the role of Rac1 in the early stage of adipogenic differentiation.

**Figure 5:**
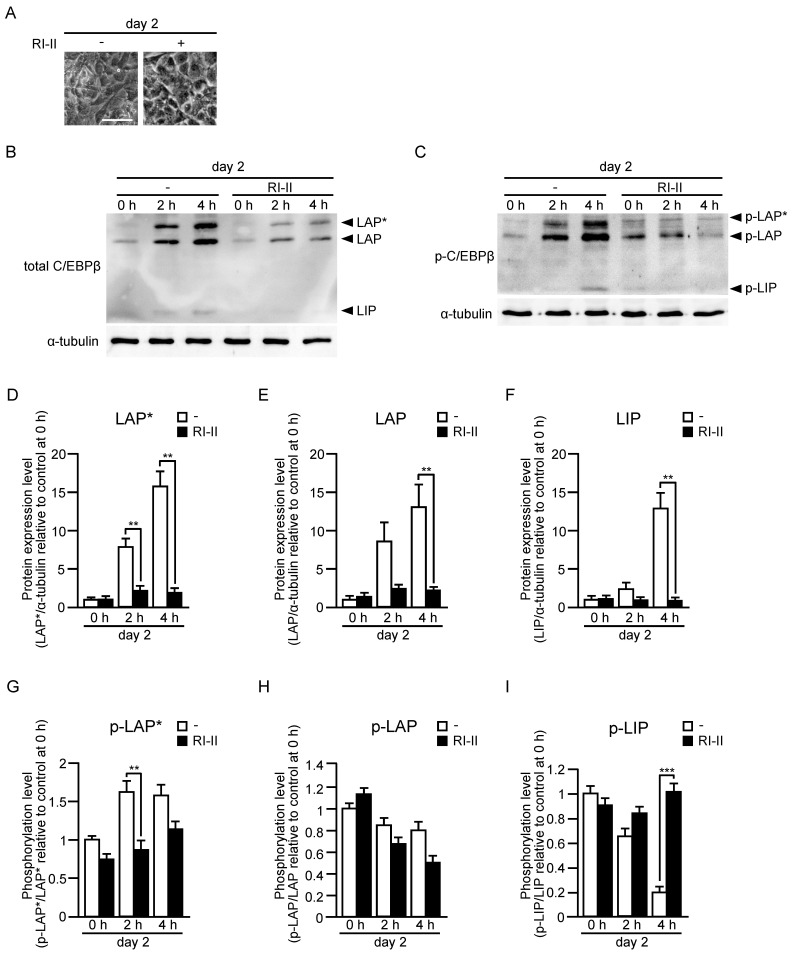
Protein and phosphorylation levels of three C/EBPβ variants in the early stage of differentiation of adipose progenitor cells into adipocytes in vitro. (**A**) Phase-contrast images of cells derived from control mice in the presence or absence of RI-II at day 2. Scale bar, 50 μm. Protein (**B**) and phosphorylation (**C**) levels of three C/EBPβ variants (LAP*, LAP, and LIP) in cells derived from control mice in the presence or absence of RI-II were determined by immunoblot analysis using antibodies against C/EBPβ (**B**) and phosphorylated C/EBPβ (**C**), respectively. α-tubulin served as a loading control. Intensities of bands for LAP* (**D**), LAP (**E**), LIP (**F**), p-LAP* (**G**), p-LAP (**H**), and p-LIP (**I**) in immunoblots were quantified. Data are shown as means ± S.E. (*n* = 3). ** *p* < 0.01, *** *p* < 0.001 (Student’s *t* test).

**Figure 6:**
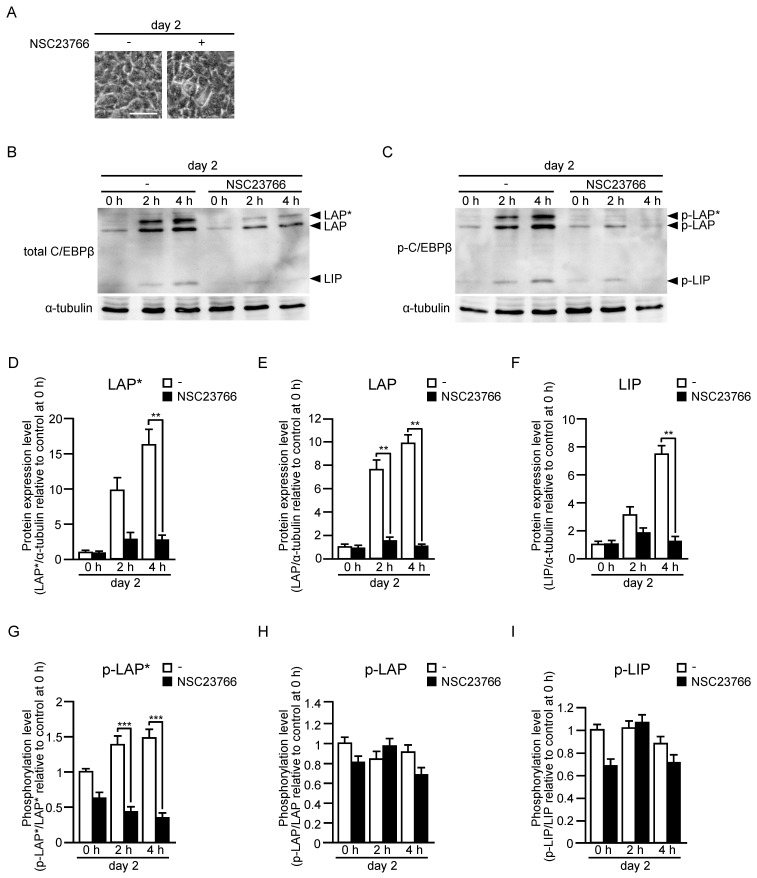
Protein and phosphorylation levels of three C/EBPβ variants in the early stage of differentiation of adipose progenitor cells into adipocytes in vitro. (**A**) Phase-contrast images of cells derived from control mice in the presence or absence of NSC23766 at day 2. Scale bar, 50 μm. Protein (**B**) and phosphorylation (**C**) levels of three C/EBPβ variants (LAP*, LAP, and LIP) in cells derived from control mice in the presence or absence of NSC23766 were determined by immunoblot analysis using antibodies against C/EBPβ (**B**) and phosphorylated C/EBPβ (**C**), respectively. α-tubulin served as a loading control. Intensities of bands for LAP* (**D**), LAP (**E**), LIP (**F**), p-LAP* (**G**), p-LAP (**H**), and p-LIP (**I**) in immunoblots were quantified. Data are shown as means ± S.E. (*n* = 3). ** *p* < 0.01, *** *p* < 0.001 (Student’s *t* test).

SVF cultures derived from control mouse WAT were treated with RI-II or NSC23766 for 24 h prior to the induction of differentiation, and further treated during differentiation. At day 2, the effect of Rac1 inhibitors on protein and phosphorylation levels of C/EBPβ was examined by immunoblot analysis (Figure 5 and Figure 6). Neither RI-II nor NSC23766 affected cell shape and the formation of lipid droplets in the cell (Figure 5A and Figure 6A). In the absence of the Rac1 inhibitor, protein levels of three C/EBPβ variants were markedly increased at day 2 (Figure 5 and Figure 6). Considering that the increase in the C/EBPβ mRNA level at day 2 was approximately twofold (Figure 3C), it is likely that the rapid increase in the protein level is ascribed to translational upregulation or inhibition of protein degradation. Both RI-II and NSC23766 almost completely inhibited differentiation-associated increase in protein levels of C/EBPβ (Figure 5 and Figure 6). Similarly to the results in the late stage of differentiation (Figure 4), Rac1 inhibition negatively affected the phosphorylation level of LAP*, but not LAP, in the early stage (Figure 5 and Figure 6). On the other hand, Rac1 inhibitors exerted almost no effect on the phosphorylation level of LIP in the early stage (Figure 5 and Figure 6), whereas knockdown of Rac1 resulted in the decreased phosphorylation level of LIP in the late stage (Figure 4).

### 2.5. Role of Rac1 in Differentiation of 3T3-L1 Cells into Adipocytes In Vitro

**Figure 7:**
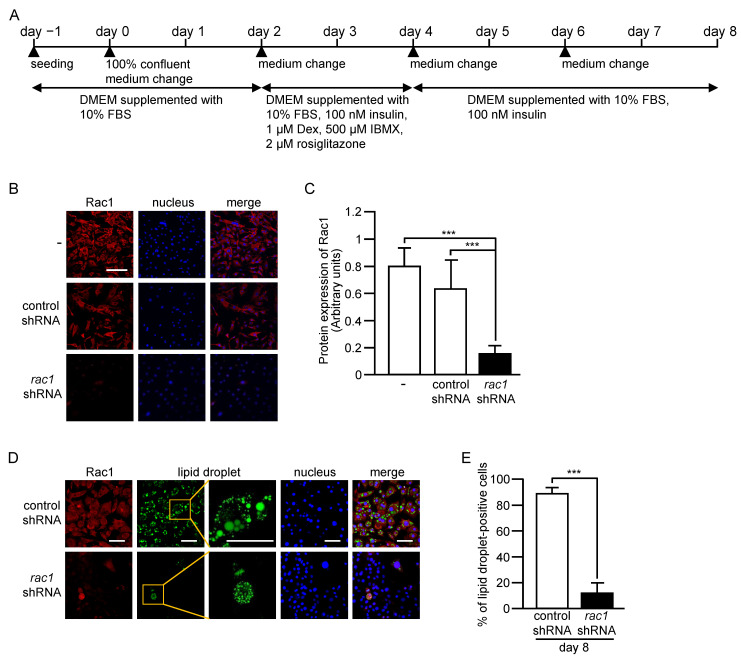
Differentiation of 3T3-L1 cells into adipocytes in vitro. (**A**) Schematic representation of the differentiation protocol. The day when cells reached confluence was referred to as day 0. (**B**) sh-RNA-mediated knockdown of Rac1 in 3T3-L1 cells. The protein expression level of Rac1 in 3T3-L1 cells that expressed control or Rac1-targeting shRNA was determined by immunofluorescent microscopy. Nuclei were stained with 4′,6-diamidino-2-phenylindole. Scale bar, 200 μm. (**C**) Fluorescent staining of Rac1 as shown in (**B**) was quantified. Data are shown as means ± S.E. (*n* = 36). *** *p* < 0.001 (Student’s *t* test). (**D**) Fluorescent staining of Rac1 and lipid droplets with an anti-Rac1 antibody and LipiDye, respectively, in differentiated 3T3-L1 adipocytes that expressed control or Rac1-targeting shRNA at day 8. Nuclei were stained with 4′,6-diamidino-2-phenylindole. For the staining of lipid droplets, the high-magnification image (right) of the boxed area in the low magnification image (left) is also shown. Scale bar, 100 μm. (**E**) The percentage of lipid droplet-containing cells in differentiated 3T3-L1 adipocytes that expressed control or Rac1-targeting shRNA at day 8 was calculated. Data are shown as means ± S.E. (*n* = 36). *** *p* < 0.001 (Student’s *t* test).

To further confirm that Rac1 plays an important role in adipogenic differentiation, we next examined the effect of Rac1 knockdown in another in vitro differentiation system using the 3T3-L1 preadipocyte line. Adipogenic differentiation of 3T3-L1 cells was induced according to a standard protocol as described previously [11,12] (Figure 7A). The day when cells reached confluence was referred to as day 0. Confluent cells were further cultured for two days in DMEM-based growth medium. At day 2, reagents, such as insulin, were added to the culture medium, inducing adipogenic differentiation (Figure 7A).

We infected 3T3-L1 cells with lentivirus expressing control or Rac1-targeting small hairpin RNA (shRNA), and puromycin-resistant cells, which expressed respective shRNAs, were selected. The expression level of Rac1 as determined by immunofluorescent microscopy was actually suppressed in 3T3-L1 cells that expressed Rac1-targeting shRNA (Figure 7B,C). Those 3T3-L1 cells that expressed control or Rac1-targeting shRNA were then subjected to the induction of adipogenic differentiation, as described above. At day 8, a large population of control shRNA-expressing cells harbored lipid droplets, which are characteristic of adipocytes (Figure 7D,E). In marked contrast, lipid droplets were detected in only a small population of cells that expressed Rac1-targeting shRNA (Figure 7D,E). Therefore, it is plausible that Rac1 is required for adipogenic differentiation of 3T3-L1 cells as well.

## 3. Discussion

In this study, we investigated the role of Rac1 in adipogenic differentiation using two different in vitro cell systems: adipose progenitor cells obtained from mouse WAT and the 3T3-L1 preadipocyte line. In both systems, functional deficiency of Rac1 led to the attenuated formation of lipid droplets within the cell, a characteristic feature of adipogenic differentiation. Therefore, it is likely that Rac1 is critically involved in the induction of adipogenic differentiation. This conclusion is consistent with our previous findings that subcutaneous and epididymal WAT in adipo-*rac1*-KO mice is significantly smaller than in control mice, showing severe atrophy [13]. Furthermore, the size of white adipocytes was reduced in adipo-*rac1*-KO mice compared with those in control mice [13].

A significant observation that needs to be considered is a difference in the adipocyte type between in vivo and in vitro experiments. Although the adipose progenitor cells used in this study were derived from WAT, adipocytes differentiated from these progenitor cells in vitro contained a number of small lipid droplets, but not a single large lipid droplet: these cells were similar in appearance to brown or beige adipocytes, rather than white adipocytes. Further characterization of adipocytes differentiated from the progenitor cells in vitro will be performed in the future. On the other hand, our results lead to the possibility that Rac1 is implicated in the differentiation into brown adipocytes as well as white adipocytes. We are currently investigating this possibility in vivo and in vitro.

We have provided evidence that the induction of various enzymes for de novo synthesis of fatty acids and triacylglycerol at the mRNA level during adipogenic differentiation largely depends on Rac1 (Figure 2). This notion was also supported by reduced mRNA levels of these enzymes observed in white adipocytes of adipo-*rac1*-KO mice in our recent study [13]. These findings are important because defects in de novo lipid synthesis due to the insufficient induction of various enzymes may account, at least in part, for the reduced size of white adipocytes and atrophy of WAT in adipo-*rac1*-KO mice.

Furthermore, we demonstrated that Rac1 contributes to the induction of two transcription factors, PPARγ and C/EBPα, which act as master switches of adipogenic differentiation [24,25] (Figure 3A,B). Towards understanding the mechanisms underlying Rac1-dependent induction of these transcription factors, we then examined the induction of upstream transcription factors—C/EBPβ and C/EBPδ (Figure 3C,D). Moreover, protein and phosphorylation levels of three variants of C/EBPβ were evaluated by immunoblot analysis both in early and late stages (Figure 4, Figure 5 and Figure 6).

In the early stage of differentiation (day 2), Rac1 was involved in the rapid increase in the protein level of LAP*, LAP, and LIP. This rapid increase in the protein level is likely to be due to the upregulation of translation or downregulation of protein degradation, given that the increase in the C/EBPβ mRNA level at day 2 was approximately twofold (Figure 3C). The precise roles of Rac1 in the regulation of translation and protein degradation of C/EBPβ remain incompletely understood, and are currently under investigation. In addition, Rac1 was responsible for the induction of C/EBPβ in the late stage (day 5–day 7) (Figure 3 and Figure 4). Rac1 may be involved mainly in transcriptional regulation in this stage, and the detailed mechanisms need to be clarified in future studies.

We demonstrated that Rac1 was involved in the induction of the mRNA level of the transcription factor SREBP-1c in the late stage of adipogenic differentiation (Figure 3E). This may also provide an explanation for the decreased expression of various enzymes for the synthesis of fatty acids and triacylglycerol (Figure 2), although the role of SREBP-1c in de novo lipogenesis in white adipocytes remains controversial [23].

Carbohydrate response element-binding proteins (ChREBPs) are also identified as major transcription factors that induce lipogenic enzymes in response to glucose in adipocytes [23]. We did not test the effect of Rac1 knockdown on the induction of ChREBPs in this study, considering that ChREBPs are mostly induced by glucose rather than insulin. However, it is possible that Rac1 knockdown causes insufficient activation of ChREBPs in vivo, because insulin-stimulated glucose uptake in white adipocytes is severely impaired in adipo-*rac1*-KO mice [13]. This possibility will be tested in our future studies.

Rac1 has been implicated in the regulation of insulin-stimulated glucose uptake in white adipocytes [10,11,12,13]. Considering that glucose is utilized for fatty acid synthesis as well as the production of ATP, defects in glucose uptake may be another major cause of the reduced size of white adipocytes in adipo-*rac1*-KO mice [13]. On the other hand, fatty acid transport from the circulation into adipocytes is also regulated by insulin [31]. Rac1 may be implicated in insulin-stimulated fatty acid uptake because insulin regulates glucose and fatty acid transport across the plasma membrane by similar mechanisms. In this case, defects in fatty acid uptake may be another cause of the impaired accumulation of lipids in white adipocytes in adipo-*rac1*-KO mice. The expression level of GPAT1, which is responsible for the synthesis of lysophosphatidic acid from glycerol-3 phosphate and fatty acids, was significantly reduced in cells derived from adipo-*rac1*-KO mice (Figure 2E). Therefore, the synthesis of triacylglycerol is expected to be impaired, at least in part, if sufficient amounts of glucose and fatty acids are incorporated from the blood. Further studies will be needed to better understand the mechanisms.

A recent study using a mouse-dedifferentiated fat cell line showed that Rac1 is involved in actin depolymerization-induced differentiation into adipocytes [32]. In particular, insulin-activated Rac1 is thought to regulate the formation of adipocyte-associated cortical actin structures, which is required for the completion of adipogenic differentiation [32]. Therefore, it is likely that Rac1 exerts multiple functions, including the regulation of glucose uptake, the expression of enzymes for lipid synthesis, and cortical actin cytoskeletal rearrangements, in developing adipocytes, and its loss may cause aberrations in these cells.

In contrast to our findings, Rac1 has been implicated in negative regulation of the expression of PPARγ and C/EBPα and the accumulation of lipid droplets in 3T3-L1 cells in response to the activation of integrins [33,34]. Thus, Rac1 may exert multiple functions in response to different stimulations in the different processes of adipogenic differentiation. It is important that the results obtained from the analysis of in vitro differentiation systems are interpreted in terms of their relevance to in vivo observations. In the present study, we revealed novel functions of Rac1 that may account for atrophy of WAT in adipo-*rac1*-KO mice, and further investigations are required to understand the mechanisms in detail.

## 4. Materials and Methods

### 4.1. Materials

A mouse monoclonal antibody against Rac1 (610650) was purchased from BD Biosciences (San Diego, CA, USA). A mouse monoclonal antibody against C/EBPβ (sc-7962) was purchased from Santa Cruz Biotechnology (Dallas, TX, USA). A rabbit polyclonal antibody against phospho-(Thr235) C/EBPβ (3084) was purchased from Cell Signaling Technology (Danvers, MA, USA). A mouse monoclonal antibody against α-tubulin (T9026) was purchased from Sigma-Aldrich (St. Louis, MO, USA). A sheep polyclonal antibody against mouse IgG (NA931) conjugated with horseradish peroxidase was purchased from Cytiva (Emeryville, MA, USA). A donkey polyclonal antibody against rabbit IgG (w4018) conjugated with horseradish peroxidase was purchased from Promega (Madison, WI, USA). A donkey polyclonal antibody against mouse IgG conjugated with CF543 (20,305) was purchased from Biotium (Fremont, CA, USA). Rac1-specific inhibitors RI-II (553511) and NSC23766 (S8031) were purchased from Merck (Darmstadt, Germany) and Selleck Chemicals (Houston, TX, USA), respectively. LipiDye (lipid droplet green, FDV-0010) was purchased from Funakoshi (Tokyo, Japan). Insulin was purchased from Eli Lilly (Indianapolis, IN, USA).

### 4.2. Animal Experiments

All animal experiments were approved by the Ethics Committee for Animal Experiments at Osaka Metropolitan University (approval codes 20-74, 20-75, 21-81, 21-82, 22-101, and 22-102) and carried out according to the institutional guidelines of Osaka Metropolitan University. All mice used in this study had a C57BL/6 genetic background. We routinely crossbred *rac1*^flox/flox^ mice [35] with adipo-*rac1*-KO mice to obtain adipo-*rac1*-KO mice for experiments. Adipoq-Cre transgenic mice [28] were used as controls throughout this study. Mice were fed a normal chow diet and adult (22- to 26-week-old) male mice were used for all experiments.

### 4.3. Conventional RT-PCR Analysis

Total cellular RNA was isolated from the SVF and mature adipocytes using the Sepasol-RNA I Super G (Nacalai tesque (Kyoto, Japan)) according to the manufacturer’s instructions. cDNAs were synthesized using the SuperScript IV first-strand synthesis system for RT-PCR (Thermo Fisher Scientific (Waltham, MA, USA)) and then amplified using KOD FX neo (Toyobo (Osaka, Japan)) and specific primers (Thermo Fisher Scientific) (5′-CTACCACGGAGACTTCTACA-3′ and 5′-ACCATAGTCTCTGAGATGGC-3′ for the *cd34* gene, 5′-AGAAGGTGGTAGAGTTCCTC-3′ and 5′-GTGTCGAGAAAGAGTGTTGG-3′ for the *perilipin 1* gene, and 5′-CTACAATGAGCTGCGTGTGG-3′ and 5′-CAACGTCACACTTCATGATGG-3′ for the *β-actin* gene) according to the manufacturer’s instructions. PCR products were analyzed by agarose gel electrophoresis.

### 4.4. Preparation of the SVF from Subcutaneous WAT

Subcutaneous WAT was excised from euthanized 22-week-old male mice. Minced subcutaneous WAT was digested in collagenase buffer (20 mM HEPES (pH 7.4), 120 mM NaCl, 5 mM KCl, 4 mM NaHCO_3_, 1 mM CaCl_2_, 0.7 mM MgSO_4_, 0.4 mM KH_2_PO_4_, and 0.3 mM Na_2_HPO_4_) supplemented with 3 mg/mL collagenase I (031-17601, Fujifilm Wako (Osaka, Japan)) at 37 °C for 1 h. Digested subcutaneous WAT was filtered through 100 μm nylon mesh to get a single-cell suspension, which was then centrifugated at 760× *g* for 10 min at room temperature. The precipitated SVF was suspended in KBM ADSC-1 (Kohjin Bio, Saitama, Japan) supplemented with 2500 IU/mL penicillin and 2500 μg/mL streptomycin and cultured at 37 °C with 5% CO_2_ to confluence.

### 4.5. Induction of Differentiation of Adipose Progenitor Cells in the SVF into Adipocytes In Vitro

The protocol for differentiation of adipose progenitor cells into adipocytes in vitro is also shown in Figure 1B. The day when cells reached confluence was referred to as day 0. At day 0, the culture medium was changed to DMEM (043-30085, Fujifilm Wako) supplemented with 10% (*v*/*v*) fetal bovine serum (FBS) (Corning, NY, USA), 2500 IU/mL penicillin, and 2500 μg/mL streptomycin, and cells were cultured for two days. The culture medium was changed to DMEM supplemented with 10% (*v*/*v*) FBS, 100 nM insulin, 1 μM dexamethasone (Dex), 500 μM 3-isobutyl-1-methylxanthine (IBMX), 2 μM rosiglitazone, 2500 IU/mL penicillin, and 2500 μg/mL streptomycin at day 2. After two days, the culture medium was changed to DMEM supplemented with 10% (*v*/*v*) FBS, 100 nM insulin, 2500 IU/mL penicillin, and 2500 μg/mL streptomycin, and cells were further cultured for two days. At day 6, the culture medium was changed again to the same medium, and cells were cultured for one more day. In some experiments, cells were treated with 25 μM RI-II or 100 μM NSC23766 from day −1.

### 4.6. Quantitative RT-PCR Analysis

Quantitative RT-PCR analysis was carried out essentially as described in [13]. Total cellular RNA was isolated from differentiating cells using the Sepasol-RNA I Super G (Nacalai tesque) according to the manufacturer’s instructions. cDNAs were synthesized using the SuperScript IV first-strand synthesis system for RT-PCR (Thermo Fisher Scientific). PCR was carried out using TB Green Premix Ex Taq II (Takara Bio (Kyoto, Japan)) and specific primers (Thermo Fisher Scientific) with Thermal Cycler Dice Real Time System III (Takara Bio) according to the manufacturer’s instructions. PCR primers are as follows: 5′-AGGTTCGTTCACTCATGGA-3′ and 5′-TCGACCAGTTTAGTTACCC-3′ for the *cre* gene, 5′-CCTGCCTGCTCATCAGTTAC-3′ and 5′-CCATAGGCCCAGATTCACTG-3′ for the *rac1* gene, 5′-GTCTACATCCTTGACTTGGC-3′ and 5′-CACTTTTGGCATCCAGGTCT-3′ for the *acly* gene, 5′-TACCTGTACAAGCAGTGTGG-3′ and 5′-CAATCCACTCGAAGACCACT-3′ for the *acc* gene, 5′-TTGCTGGCACTACAGAATGC-3′ and 5′-CTCAGAGCGACAATATCCAC-3′ for the *fasn* gene, 5′-GAGTACGTCTGGAGGAACAT-3′ and 5′-AGAGCGCTGGTCATGTAGTA-3′ for the *scd1* gene, 5′-GCTGGGTGTTACTAAAGCTC-3′ and 5′-GTCAATGTGGGATCTGTGCA-3′ for the *gpat1* gene, 5′-AGCATCAGGCTTCCACTATG-3′ and 5′-TGGATCCGGCAGTTAAGATC-3′ for the *pparγ* gene, 5′-TGGACAAGAACAGCAACGAG-3′ and 5′-GGTCATTGTCACTGGTCAAC-3′ for the *c/ebpα* gene, 5′-TGAGCGACGAGTACAAGATG-3′ and 5′-AGCTGCTTGAACAAGTTCCG-3′ for the *c/ebpβ* gene, 5′-GAGCGCAACAACATCGCTGT-3′ and 5′-CGCTGATGCAGCTTCTCGTT-3′ for the *c/ebpδ* gene, 5′-AACACTGTGACCTCACAGGT-3′ and 5′-CTCCTGCATCTGTCTTCACA-3′ for the *srebp1c* gene, and 5′-ATGAAGATCAAGATCATTGCTCCTC-3′ and 5′-ACATCTGCTGGAAGGTGGACAG-3′ for the *β-actin* gene. Relative mRNA levels were determined by the ΔΔCt method followed by normalization with the β-actin mRNA level.

### 4.7. Immunofluorescent Microscopy

Immunofluorescent microscopy was carried out essentially as described in [13]. Cells were fixed with 40 mg/mL paraformaldehyde in phosphate-buffered saline (PBS) for 30 min. Rac1 was detected with anti-Rac1 and fluoresceinated secondary antibodies. Lipid droplets and nuclei were stained with LipiDye and 4′,6-diamidino-2-phenylindole, respectively. Images were obtained and analyzed using a confocal laser-scanning microscope (FV1200, Olympus, Tokyo, Japan). Fluorescent intensities were quantified using ImageJ software.

### 4.8. Immunoblot Analysis

Immunoblot analysis was carried out essentially as described in [13]. Proteins separated by sodium dodecyl sulfate–polyacrylamide gel electrophoresis were transferred onto a 0.45 μm-pore polyvinylidene difluoride membrane (Cytiva, Shanghai, China). Membranes were incubated with primary antibodies, and then horseradish peroxidase-conjugated secondary antibodies. Specific proteins were visualized by Chemi-Lumi One Ultra (Nacalai tesque). Images were captured, and densitometric analysis was carried out using a chemiluminescence imaging system (Ez-Capture MG, Atto, Tokyo, Japan).

### 4.9. Induction of Differentiation of 3T3-L1 Cells into Adipocytes In Vitro

Induction of differentiation of 3T3-L1 cells into adipocytes in vitro was carried out essentially as described in [12]. The protocol for differentiation of 3T3-L1 cells into adipocytes in vitro is also shown in Figure 7A. Undifferentiated 3T3-L1 cells were cultured in DMEM supplemented with 10% (*v*/*v*) FBS, 100 IU/mL penicillin, and 100 μg/mL streptomycin. The day when cells reached confluence was referred to as day 0. At day 0, the culture medium was changed to the same medium, and cells were cultured for two more days. The culture medium was changed to DMEM supplemented with 10% (*v*/*v*) FBS, 100 nM insulin, 1 μM Dex, 500 μM IBMX, 2 μM rosiglitazone, 100 IU/mL penicillin, and 100 μg/mL streptomycin at day 2. After two days, the culture medium was changed to DMEM supplemented with 10% (*v*/*v*) FBS, 100 nM insulin, 100 IU/mL penicillin, and 100 μg/mL streptomycin, and cells were further cultured for two days. At day 6, the culture medium was changed again to the same medium, and cells were cultured for two more days.

### 4.10. shRNA-Mediated Knockdown of Rac1 in 3T3-L1 Cells

The TRC2-pLKO1-puro plasmid containing shRNA for mouse Rac1 (GGAGACGGAGCTGTTGGTAAA, TRCN0000310888) and the nonmammalian shRNA control plasmid (TRC2-pLKO.5-puro non-target shRNA #1) (SHC202) were purchased from Sigma-Aldrich. Either one of these shRNA expression lentiviral plasmids was introduced into HEK-293TN cells with lentiviral packaging plasmids (pMISSION GAG POL and pMISSION VSV-G) using the TransIT-293 Reagent (Takara Bio). Forty-eight hours later, the culture medium containing lentiviruses was collected and then filter-sterilized. 3T3-L1 cells were infected with the lentiviruses at a multiplicity of infection of 4000 in the culture medium supplemented with 7 μg/mL polybrene. Those 3T3-L1 cells that stably expressed shRNA were selected with 2 μg/mL puromycin for three days.

## Data Availability

The data presented in this study are available on request.

## Abbreviations

ACC	acetyl-CoA carboxylase
ACLY	ATP citrate lyase
adipo-*rac1*-KO	adipocyte-specific *rac1* knockout
bZIP	basic leucine zipper
C/EBP	CCAAT/enhancer-binding protein
ChREBP	carbohydrate response element-binding protein
Dex	dexamethasone
DMEM	Dulbecco’s modified Eagle’s medium
FASN	fatty acid synthase
FBS	fetal bovine serum
GPAT1	glycerol-3-phosphate acyltransferase 1
IBMX	3-isobutyl-1-methylxanthine
PBS	phosphate-buffered saline
PI3K	phosphoinositide 3-kinase
p-LAP*	phosphorylated LAP*
p-LAP	phosphorylated LAP
p-LIP	phosphorylated LIP
PPARγ	peroxisome proliferator-activated receptor γ
RT-PCR	reverse transcriptase-polymerase chain reaction
SCD1	stearoyl-CoA desaturase 1
shRNA	small hairpin RNA
SREBP-1c	sterol regulatory element-binding protein 1c
SVF	stromal-vascular fraction
WAT	white adipose tissue
